# Additive Effects of Glutathione in Improving Antibiotic Efficacy in HIV–*M.tb* Co-Infection in the Central Nervous System: A Systematic Review

**DOI:** 10.3390/v17010127

**Published:** 2025-01-17

**Authors:** Leena Nabipur, Michael Mouawad, Vishwanath Venketaraman

**Affiliations:** College of Osteopathic Medicine of the Pacific, Western University of Health Sciences, Pomona, CA 91766, USA; leena.nabipur@westernu.edu (L.N.); michael.mouawad@westernu.edu (M.M.)

**Keywords:** HIV, tuberculosis, co-infection, glutathione, immune modulation, central nervous system

## Abstract

Background: HIV and tuberculosis (TB) co-infection poses a significant health challenge, particularly when involving the central nervous system (CNS), where it leads to severe morbidity and mortality. Current treatments face challenges such as drug resistance, immune reconstitution inflammatory syndrome (IRIS), and persistent inflammation. Glutathione (GSH) has the therapeutic potential to enhance treatment outcomes by improving antibiotic efficacy, reducing inflammation, and mitigating immune dysfunction. Methods: Relevant studies were identified through systematic searches of PubMed, Elsevier, WHO, and related databases. Inclusion criteria focused on preclinical and clinical research examining GSH or its precursors in HIV, TB, or co-infection, with emphasis on microbial control, immune modulation, and CNS-related outcomes. Results: Preclinical studies showed that GSH improves macrophage antimicrobial function, reduces oxidative stress, and limits *Mycobacterium tuberculosis* (*M.tb*) growth. Animal models demonstrated reduced bacterial burden in the lungs, liver, and spleen with GSH supplementation, along with enhanced granuloma stability. Clinical studies highlighted increased TH1 cytokine production, reduced inflammatory markers, and improved CD4+ T cell counts in HIV–*M.tb* co-infected patients. N-acetylcysteine (NAC), a GSH precursor, was shown to significantly enhance the efficacy of first-line TB antibiotics and mitigate treatment-associated toxicity. Discussion: GSH shows promise as an adjunct therapy for HIV–*M.tb* co-infection, particularly for cases involving the CNS, where it may improve immune recovery and reduce inflammation. However, evidence is limited by small sample sizes and a lack of randomized trials. Future research should focus on developing CNS-directed GSH formulations and evaluating its integration into current treatment protocols to address the dual burden of HIV and TB, ultimately improving patient outcomes.

## 1. Introduction

Human immunodeficiency virus (HIV) and tuberculosis (TB) continue to pose significant global health challenges. According to the World Health Organization (WHO), in 2023 there were around 39.9 million people living with HIV worldwide. Among these individuals, there were an estimated 630,000 people who died from HIV-related causes, and another 1.3 million individuals who newly acquired the disease [1]. Although HIV is not a curable disease, it can be managed as a chronic condition with antiretroviral therapy (ART). HIV suppresses the immune system, allowing greater susceptibility to opportunistic infections, most notably tuberculosis (TB), and those infected with HIV are at a significantly higher risk of being infected by *Mycobacterium tuberculosis* (*M.tb*) compared to the general population. In people living with HIV (PLWH), TB is currently the leading cause of death; in 2023 the total number of deaths by TB was 1.25 million with 161,000 of those individuals having HIV [2].

HIV can lead to immunosuppression characterized by the depletion of CD4+ T cells, macrophage dysfunction, and impairment of the blood–brain barrier. These together can increase the risk of opportunistic infections by organisms like *M.tb*. This enables greater entry of both HIV and *M.tb* into the central nervous system (CNS) [3,4]. HIV-induced immunosuppression can also lead to the reactivation of latent *M.tb* infection [5]. The most common form of active *M.tb* infection is pulmonary tuberculosis (PTB), as the disease is initially inhaled and transmitted through aerosol droplets and thus infects the lungs. In the lungs, the bacteria are phagocytized by alveolar macrophages, which lead to the development of granulomas in an attempt to control the bacilli, a hallmark of *M.tb* infection. Granulomas aim to control spread of the bacilli extrapulmonary regions of the body [6]. However, in those that are immunocompromised, the immune system may not be able to adequately control *M.tb*. This can lead to miliary TB, indicating dissemination of *M.tb* to other organs, including CNS involvement which can manifest as tuberculosis meningitis (TBM) and tuberculomas. Both of these forms of TB are associated with significant morbidity and mortality [7].

HIV–*M.tb* co-infection in the CNS has a poor prognosis and is associated with high morbidity and mortality. It is clinically significant due to its complex clinical manifestations. Both pathogens can lead to CNS disease, HIV through HIV-associated neurocognitive disorders (HAND), and TB through CNS tuberculosis. The synergistic impact of the co-infection of both pathogens not only accelerates disease progression, but it can also complicate diagnosis and treatment strategies [4,7,8]. There are unique challenges in the treatment of HIV–*M.tb* co-infection, particularly when there is involvement of the CNS. Current HIV treatment involves the use of ARTs, and first line therapy for TB is isoniazid (INH), rifampin (RIF), ethambutol (EMB), and pyrazinamide (PZA). This current treatment regimens has its limitations due to the potential for drug–drug interactions, possible adverse effects, and the development of drug-resistant *M.tb* strains [7].

Glutathione (γ-glutamylcysteinylglycine, GSH) is an important antioxidant, playing a significant role not only in redox homeostasis but also in immune function. Specifically, it serves as a key part of the innate immunity against *M.tb* infection. However, in those with HIV, there are decreased levels of GSH, impairing the immune system’s ability to control *M.tb*. The connection between HIV depletion of GSH and its role in TB highlights its potential in the treatment of HIV–*M.tb* co-infection, potentially enhancing current treatment regimens while also helping reduce inflammation [9]. Furthermore, GSH supplementation has been shown to protect HIV patients against opportunistic diseases by reducing oxidative stress and chronic inflammation in HIV-associated neurodegenerative diseases, enhancing Th17 responses in HIV patients who have weakened gut mucosal defenses, and restoring Th1 responses to improve resistance to TB co-infection [10].

The aim of this study is to explore the potential of GSH in treating HIV–*M.tb* co-infection. Specifically, we are interested in its ability to increase antibiotic therapy efficacy while also mitigating significant and chronic inflammation in HIV–*M.tb* co-infection of the CNS. This systematic review aims to synthesize evidence on the therapeutic potential of glutathione in HIV–*M.tb* co-infection of the CNS. We hope the results of this study provide insight into new possibilities with regard to therapeutic strategies to improve clinical outcomes for affected individuals.

## 2. Materials and Methods

**Figure 1:**
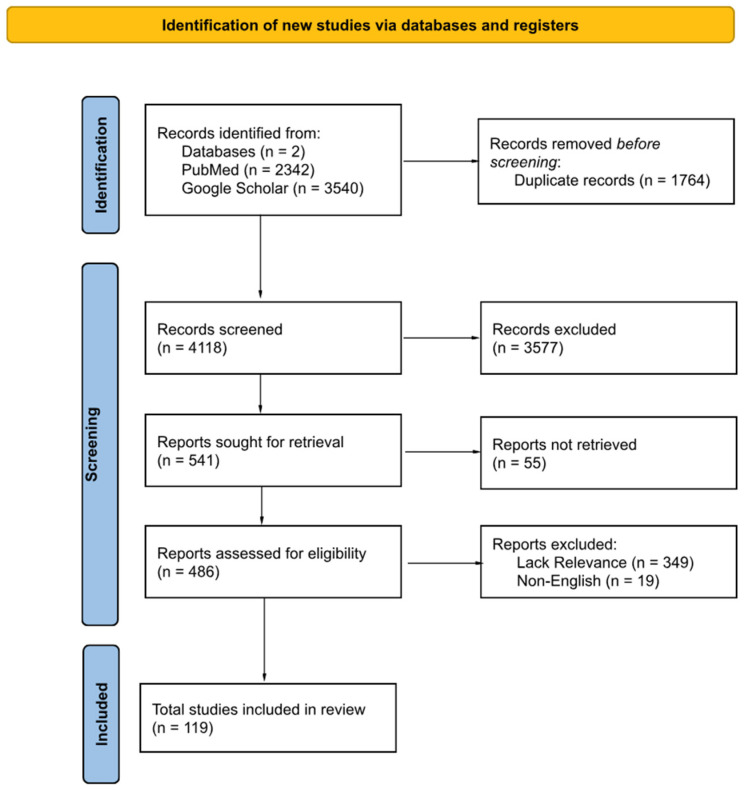
Identification of studies via databases using PRISMA flow diagram.

A comprehensive literature search was conducted through PubMed and Google Scholar databases, and related platforms, to identify relevant studies examining glutathione (GSH) or its precursors in the context of HIV, tuberculosis (TB), or HIV-TB co-infection. Search terms included “glutathione”, “GSH”, “HIV infection”, “tuberculosis”, “co-infection”, “immune modulation”, and “central nervous system”. Boolean operators (AND/OR) were used to construct the search strings, and filters were applied to include only peer-reviewed English-language articles published up to 30 December 2024. Titles and abstracts were independently screened by two reviewers to assess eligibility based on predefined inclusion criteria, such as relevance to HIV, TB, or CNS co-infection and investigation of GSH’s role in immune modulation or oxidative stress. Full-text articles were reviewed, and disagreements were resolved through discussion or consultation with a third reviewer. Figure 1 provides a PRISMA flow diagram illustrating the selection process, which began with 5882 records, of which 1764 were excluded during the title/abstract screening phase. Ultimately, 119 studies were included for analysis. Extracted information included study characteristics, population demographics, interventions, and outcomes assessed. Studies lacking relevance to targeted diseases and non-English articles were excluded. Selection bias was minimized by clearly defining inclusion and exclusion criteria and maintaining a detailed record of the study screening and selection process, including reasons for exclusions. A comprehensive search was conducted across multiple databases to ensure diverse and representative sources were included. Data were extracted, summarizing interventions, key findings, and limitations of research. Findings from preclinical and clinical research were synthesized to find recurring themes, therapies, and gaps in current knowledge, emphasizing GSH’s role in improving outcomes in HIV–*M.tb* co-infection. We have registered the protocol for this systematic review with the Open Science Framework (OSF) to enhance transparency and reproducibility. The registration can be accessed at https://doi.org/10.17605/OSF.IO/R5XC3.

## 3. Results

### 3.1. HIV and M.tb Interaction in the CNS

The blood–brain barrier (BBB) is a semipermeable barrier composed of human brain microvascular endothelial cells (HBMECs). HBMECs compose the lining of the walls of brain capillaries [11]. They function as a barrier in order to separate the bloodstream from the cerebrospinal fluid (CSF) and protects the CNS from foreign substances in the blood, such as various infectious viruses and bacteria [12].

HIV can compromise the BBB, leading to the CNS involvement of HIV and *M.tb.* There are several ways that this is accomplished. HIV can alter mitochondrial function in HBMECs via disruption of membrane potential, promoting oxidative stress through ROS, and reducing mitochondrial expression via upregulation of hypoxia-inducible factor 1-alpha [13,14]. HIV also possesses several viral proteins, one of which is the HIV protein transactivator of transcription (Tat). Free Tat is secreted by various HIV-infected immune cells, such as monocytes, macrophages, microglia, astrocytes, and CD4+ T cells. Free Tat has the ability to cross the BBB while extracellular vesicle Tat cannot [15,16]. HIV Tat works to disrupt the BBB through multiple mechanisms. This includes an increased ER stress and the promotion of the unfolded protein response to cause apoptosis of HBEMCs [16,17]. Additionally, Tat can activate nuclear factor-кB (NF-кB) and activator protein-1 (AP-1) while also increasing the secretion of proinflammatory cytokines TNF-α, IL-6, and IL-8. These combined effects lead to inflammation, enhancing the recruitment of monocytes, therefore exacerbating BBB damage. This inflammatory state also leads to increased levels of matrix metalloproteinases. These enzymes break down the extracellular matrix of HBEMCs, furthering BBB compromise [18].

Once HIV infects monocytes, they are then able to cross the BBB and release inflammatory cytokines [19]. This can be further exacerbated by another HIV viral protein, HIV nef, that stimulates the release of IL-1β, which further increases BBB permeability [20]. HIV infection also downregulates key tight junction proteins, such as ZO-1, a key component of the BBB [21]. HIV infection has the ability to induce autophagy and increase the levels of NO through stimulation of eNOS and iNOS, leading to further damage of HBEMCs [16,22]. Another HIV viral protein, gp120, along with HIV Tat both promote oxidative stress by increasing NADPH oxidase activity and reducing antioxidant enzymes such as GSH peroxidase, resulting in elevated reactive oxygen species (ROS) levels [23].

*M.tb* is spread through respiratory droplets and infection is usually localized to the lungs. However, in immunocompromised individuals, such as those with HIV, it can spread to the bloodstream leading to miliary TB, and even CNS involvement [24]. *M.tb* CNS involvement begins with caseous granulomas called tuberculomas or Rich Foci. Tuberculomas have coalescent growth and caseous necrosis and are a combination of inflammatory cells such as lymphocytes and macrophages. They form as the immune system attempts to contain *M.tb.* However, they can fail and rupture, releasing bacilli into the subarachnoid space contributing to the development of TBM [25,26]. Other potential mechanisms of entry into the CNS is through the “Trojan Horse” method of being trafficked across the BBB in infected immune cells such as macrophages and neutrophils, invasion of HBMECs through reorganization of actin, and endothelial adhesion through the pknD virulence factor [27].

### 3.2. HIV Pathogenesis: Mechanism of CD4+ T Cell Depletion

HIV specifically infects CD4+ T helper lymphocytes. HIV infection of these cells starts with the attachment to the CD4 receptor and one of the co-receptors, CXCR4 or CCR5, on a CD4+ T cell [28]. HIV viral envelope proteins gp120 and gp41 bind the CD4 receptor inducing a conformational change that allows the gp41 subunit to fuse the HIV viral and CD4+ T cell membranes [29,30,31]. Following membrane fusion, the viral genome and enzymes such as reverse transcriptase, integrase, and protease, are transported into the CD4+ T cell, where replication can take place and the viral DNA can be integrated into the host genome [32]. The integrated viral DNA stays dormant until activated, while activation leads to viral assembly and release which lyses the host cell in the process [33,34,35].

There are several mechanisms that lead to CD4+ T cell depletion in HIV infection. The first is through direct infection and cell lysis as described above. Additionally, uninfected CD4+ T cells called bystander cells can undergo apoptosis from infected cells through secretion of inflammatory signals, cytokines, or inhibitory molecules [36]. HIV Tat protein has also been shown to play a role in T cell depletion. The proposed mechanisms are the secretion of TGF-B1 to inhibit T-lymphopoiesis at the level of the bone marrow and also through mediating apoptosis in the periphery [37,38]. Another way that T cells are inhibited in chronic HIV infection is through a process called T cell exhaustion. This occurs due to continuous immune stimulation that can lead to the increased expression of immune checkpoint inhibitors such as PD-1, resulting in a blunted T cell response and loss of control of HIV infection [39,40]. HIV not only affects T cells but also impairs the phagocytic function of macrophages; this contributes to HIV infection by acting as a reservoir for the virus [41,42].

### 3.3. Mycobacterium tuberculosis Immune Evasion

As previously noted, *M.tb* infects the lungs first. It is transmitted through respiratory droplets and infects alveolar macrophages and the lung epithelial cells, with alveolar macrophages being the primary cell target of *M.tb* in the first two weeks of infection. After this period, the infected alveolar macrophages spread out of the alveoli and enter the lung interstitium, where they can infect other macrophage types [6].

Granulomas are a key part of the host immune defense and the main setting of pulmonary TB control. These structures consist of various immune cells such as macrophages, neutrophils, and lymphocytes and depend on CD4+ T cells for assembly and function. This reliance on CD4+ T cells highlights how those with HIV can have increased *M.tb* susceptibility. A fibrotic granuloma appearance indicates proper immune control of *M.tb* and caseous granulomas are indicative of poor *M.tb* control [43]. On the other hand, granulomas can also provide a protective niche by restricting CD4+ T cells away from the core and inhibiting Th1 cells through IL-10 mediated suppression [44,45]. Additionally, GSH and its derivative S-ntitrosoglutathione (GSNO) plays an important role in controlling *M.tb* infection and is a key factor in host immune response, specifically within macrophages. GSH limits intracellular growth of *M.tb* and when macrophages are activated, GSNO is formed and suppresses bacterial replication [9].

There are several ways in which *M.tb* evades host immune defenses and creates niches where it can survive and proliferate. *M.tb* prevents degradation from macrophage destruction by its ability to coat itself with antacid, 1-TbAd, and inhibit fusion with lysosomes [46]. Additionally, infected alveolar macrophages generate a nuclear factor erythroid 2-related factor 2 (NRF2)-driven antioxidant transcriptional response, causing a decrease in inflammation, protecting the bacilli [47]. While residing in alveolar macrophages, *M.tb* can access the host cell’s iron and fatty acid supplies while simultaneously avoiding excessive oxidative stress, creating a permissive environment [48]. *M.tb* possesses catalase–peroxidase, allowing the detoxification of ROS [13]. In addition, it possesses proteins such as NdKA, CpsA, and PPE2 that can block the production of NADPH oxidase-mediated ROS. This allows survival within macrophages and prevents autophagy. Another method of autophagy inhibition is through its proteins PE-PGRS, which suppress MHC class II antigen presentation [49,50]. Lastly, protein kinase G (PknG) is able to suppress the host NF-κB signaling and immune response by binding and conjugating host ubiquitin (Ub) at a lysine residue, leading to its release and transfer [51]. Another way that *M.tb* evades the immune system is by disrupting GSH synthesis and therefore impairing GSNO production. This leads to a decrease in macrophage ability to control the infection [9].

### 3.4. Pathogenesis and Clinical Manifestations of CNS Involvement

Around half of those infected with HIV experience some form of HIV-associated neurocognitive disorder (HAND) [52]. HAND is classified into three levels of severity: asymptomatic neurocognitive impairment (ANI), mild neurocognitive impairment (MNI), and HIV-associated dementia (HAD). The most severe form, HAD, is characterized by significant motor and memory loss and thus a poor prognosis [53]. The loss of the function and protection of the blood–brain barrier (BBB) through previously described mechanisms is believed to be a key factor in the development of HAND, allowing HIV to spread to the CNS.

The most common symptom of HAND is cognitive dysfunction, often including impairments in processing speed, attention, memory, and executive function. Although rarer, some patients may experience sensory–perceptual impairments [54]. HIV can lead to HAND through various mechanisms. BBB disruption allows HIV proteins and infected immune cells, such as monocytes, to cross into the CNS. Once inside, infected perivascular macrophages promote the formation of giant cells and thus HIV encephalitis [55]. Various HIV neurotoxic proteins, including gp120 and gp41, contribute to the development of HAND through axonal and neuronal damage by oxidative stress and apoptosis via the production of ROS and NO-dependent mechanisms [56,57]. Additionally, HIV Tat can aid in the development of HAND through its previously described effects on the BBB. It also induces neurotoxicity through proinflammatory cytokine stimulation, such as TNF-alpha and excitotoxicity, leading to apoptosis of neurons [57]. Furthermore, the polymorphisms of Tat, the CC motif and R57 residue, can lead to neural inflammation through the recruitment of macrophages and monocytes into the brain [38]. Lastly, the previously mentioned disruption of activity of various antioxidant enzymes, including GSH peroxidase, results in increased oxidative stress and neuronal injury [58].

CNS involvement of TB can manifest in several ways, including TBM and CNS tuberculomas. Symptoms in those with TBM usually begin with nonspecific prodrome features such as fever, night sweats, and intermittent headaches. This is then followed by a meningitis phase that involves persistent headaches, vomiting, confusion, coma, seizures, multiple cranial nerve deficits, and meningismus symptoms [59,60]. Tuberculomas are lesions that occupy space and are spread through the bloodstream until they make their way to the brain parenchyma. They are similar to tuberculous abscesses but are smaller and without pus-filled centers [61]. They often occur in those already infected with TBM. Tuberculomas have several stages of evolution, and the most active form is when it is caseous. In MRIs, they typically show a T1W and T2W hypointense center, although some liquified areas may show a hyperintense T2W [62]. The majority of CNS TB involvement is in the form of TBM. It arises after dissemination from pulmonary TB or from latent disease reactivation [63,64]. The bacilli spreads to the brain via Rich Foci rupture in the subarachnoid space. This results in arachnoiditis at the base of the brain and optic chiasm, leading to entrapment of multiple cranial nerves. There can also be arteritis and phlebitis in this region leading to infarcts due to vasospasm, thrombosis, or hemorrhage. Furthermore, in patients who have prolonged inflammation, hydrocephalus can occur either through disruption of circulation and absorption of CSF or through obstruction due to a narrowing of the cerebral aqueduct and possibly even a tuberculoma [59].

The non-specific symptoms, symptom overlap, and varied presentations of both *M.tb* and HIV CNS involvement can make diagnosis difficult and lead to potential delays in detection.

### 3.5. Role of Glutathione in Cellular Defense

Glutathione is a tripeptide composed of glutamate, cysteine, and glycine found in most mammalian cells [65,66,67]. It serves as a key antioxidant, maintaining redox homeostasis while playing vital roles in cell cycle regulation, apoptosis, immunological defense, and reducing pathological irregularities. Redox equilibrium ensures that physiological, non-toxic levels of ROS are present in cells, with oxidative stress occurring if production exceeds the capacity of antioxidant defense [68]. GSH is a biological buffer, in which its antioxidant properties are important for neutralizing elevated ROS and RNS. Low GSH levels are a common pathway influencing all risk factors [10,69,70]. In a reaction catalyzed by GSH peroxidase, two GSH molecules form a disulfide bond through sulfhydryl dehydrogenation, resulting in the production of glutathione disulfide (GSSG). Then, GSH reductase oxidizes NADPH and converts GSSG back into GSH. In conditions of high oxidative stress, there is a reduction in cellular uptake or synthesis of cysteine. This causes GSSG content to rise, which causes the ratio of GSH to GSSG to decrease, thus reflecting a poorer redox capacity of the cell [63,71,72]. Additionally, GSH is involved in the metabolism of endogenous (i.e., estrogens, leukotrienes, and prostaglandins) and exogenous (i.e., drugs) compounds, suggesting a molecular basis for detoxification processes that eliminate foreign substances from the body [73].

Beyond its redox activity, GSH is critical in sustaining signaling pathways and maintaining mitochondrial function, both of which are key for cell survival and homeostasis. Mitochondria rely on GSH to protect against damage from cellular stresses. Disruptions in GSH levels within mitochondria can lead to impaired energy production and cell death [63]. In the immune system, GSH is involved in T cell activation, cytokine production and proper functioning of antigen-presenting cells (APCs) such as macrophages and dendritic cells. GSH is particularly important for dendritic cells, influencing antigen uptake, and cytokine release. Alterations in the functionality of these cells due to low GSH levels contribute to heightened inflammation and exacerbation of disease progression [74]. GSH also promotes the balance between regulatory T cells and effector T cells, which is crucial for preventing excess activation of the immune system as well as maintaining tolerance.

In the context of HIV, GSH diminishment is a hallmark of the disease. HIV-infected individuals have deficiencies of intracellular GSH in red blood cells (RBCs), T cells, NK cells, and monocytes [75,76,77]. Furthermore, it has been demonstrated that the levels of GSH are significantly reduced in brain tissue samples derived from the frontal cortex of individuals with HIV infection [78]. Additionally, the GSH composition found in macrophages from HIV-positive subjects heavily favored GSSG, which lacks antioxidant activity [79]. Low levels of intracellular GSH in macrophages were observed in patients with untreated HIV and are associated with poorer outcomes [5,80]. Additionally, the overproduction of proinflammatory cytokines, as seen in IL-1, IL-17, and TNF-alpha, in HIV-positive individuals led to increased production of free radicals as well as decreased expression of genes coding for enzymes responsible for de novo synthesis of GSH in macrophages [5]. These underlie the reasoning of why HIV patients have a loss of immune function.

In TB, GSH plays a role in the host’s ability to contain and control the pathogen. Macrophages are the main phagocytic cells involved in controlling *M.tb* infection. Activation of macrophages leads to the production of antimicrobial molecules including ROS and RNIs [81,82,83,84]. Macrophages rely on adequate GSH levels to sustain antimicrobial functions and protect against the toxic effects of the generation of ROS and RNI. GSH can modulate inflammation by reducing oxidative stress and limiting the excessive production of proinflammatory cytokines, which can drive tissue damage and granulomatous inflammation. Studies suggest that increasing GSH levels in macrophages can improve their capacity to restrict *M.tb* growth and support infection resolution [85,86]. Mycobacteria rely on mycothiols to regulate their redox activity, as they do not produce GSH [87]. Exposure to high concentrations of GSH can disrupt the redox balance in mycobacteria, which depend on mycothiol as an alternative thiol for redox regulation, ultimately inhibiting growth. Like macrophages, natural killer cells (NK cells) also play an important role in the innate defense against *M.tb* infection [88,89,90]. These larger lymphocytes express both CD16 and CD56 and have surface ligands that are capable of inducing apoptosis [88,89,90,91]. Previous studies have demonstrated that low GSH levels impair the cytolytic activity of NK cells. However, when both NK cells and macrophages work together, they can effectively combat intracellular M tb infection. GSH plays a role in enhancing NK cell function, thereby aiding in the control of M tb infection within macrophages. Earlier studies indicated that individuals infected with HIV have significantly lower levels of GSH within their T cells in comparison to healthy subjects, and this decrease correlated with reduced production of Th1 cytokines and compromised control of *M.tb* infection. However, a limitation of this and other studies is that it was not specified whether the patients studied were receiving effective antiviral therapy, and for how long [89].

**Figure 2:**
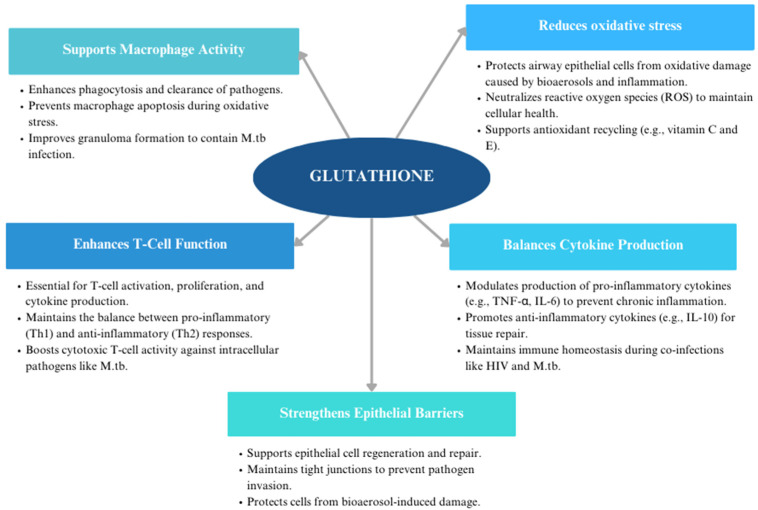
The role of glutathione (GSH) in immune modulation and defense.

When HIV and *M.tb* co-infect, these pathogens exacerbate oxidative stress and inflammation, resulting in significant neuropathology. In this context, GSH diminishment contributes to a heightened inflammatory state, impaired immune responses, and a reduced ability of host cells to manage oxidative stress. A decrease in the amount of reduced forms of GSH as seen in HIV infected patients correlated with an increase in the growth of *M.tb* in macrophages [79]. T lymphocytes derived from HIV infected individuals have low levels of GSH, which correlates with decreased levels of TH1 cytokines (IL-2, IL-12, IFN-y) and enhanced growth of *M.tb* inside human macrophages [90]. Additionally, GSH levels are decreased significantly in NK cells in HIV infected subjects, which correlates with a considerable increase in growth of *M.tb* inside monocytes [88]. This altered environment facilitates microbial persistence and reduces the efficacy of antibiotics and antiretrovirals. However, the results of studies show that restoring the levels of GSH in HIV-positive patients reverts the loss of innate immune functions in macrophages. ROS production was mitigated, and GCLC increased in number while GSR was decreased, indicating how conditions of oxidative stress were reduced through the supplementation of GSH [79]. The effects of GSH were seen when immune cells from HIV positive subjects were treated with a GSH-enhancing agent (NAC/L-GSH), which showed improvement in control of intracellular *M.tb* infection [77]. By restoring GSH levels, it may be possible to improve the efficacy of these treatments, enhance immune recovery, and mitigate inflammation within the CNS. Figure 2 illustrates the roles of glutathione in immune defense, including macrophage activity, oxidative stress reduction, cytokine regulation, T cell function, and epithelial barrier maintenance.

### 3.6. Additive Effects of Glutathione in HIV–M.tb Co-Infection

Treating individuals co-infected with HIV and *M.tb* is challenging due to the known interaction between antituberculosis and highly active antiretroviral therapy (HAART). The use of multiple drugs in treatment protocols lowers mortality, but can lead to drug–drug interactions and the development of immune reconstitution inflammatory syndrome (IRIS) [92]. IRIS occurs when immune function begins to recover after the initiation of highly active HAART, typically within the first six months [93]. This recovery, marked by increasing CD4+ T cell counts, can trigger an excessive inflammatory response to preexisting infections [92]. While HAART generally improves survival, IRIS can cause significant complications. In individuals co-infected with HIV and TB, it may present as tuberculous lymphadenitis, skin lesions, peritonitis, or bowel perforations, often complicating the clinical course despite effective HIV treatment. Low CD4+ T cell counts before ART initiation (<50–100 cells/μL) which is then followed by a CD4+ T cell raise is an important risk factor for developing IRIS [92,93,94,95,96]. Another risk factor is starting TB treatment soon after the initiation of HAART [97,98]. Extrapulmonary TB has also been reported to be associated with a higher risk of TB-IRIS [99,100]. Findings from clinical trials indicate that supplementation with liposomal glutathione (L-GSH) in HIV-infected individuals restored redox homeostasis, induced a cytokine balance, and improved immune responses against *M.tb* infection [101]. N-Acetyl Cysteine (NAC), a GSH improving agent, has been used, limited to the setting of acetaminophen toxicity. However, recent studies have also discovered an immunomodulatory effect of the drug [77,102,103].

NAC is a promising adjunct therapy, as evidence indicates it can reduce mortality associated with isoniazid (INH) and rifampin (RIF) induced hepatitis. One study found that combining NAC with antituberculosis antibiotics (INH, RIF, ethambutol EMB, or pyrazinamide PZA) significantly reduced intracellular *M.tb* survival. NAC paired with INH achieved complete bacterial clearance, outperforming INH alone, while enhancing macrophage function and reducing proinflammatory cytokines. RIF’s efficacy improved with NAC, dramatically lowering bacterial loads and reducing TNF-α and IL-10, improving immune homeostasis. NAC with ethambutol reduced bacterial survival 4-fold and increased macrophage antimicrobial activity. PZA combined with NAC enhanced phagosome maturation and pathogen elimination, achieving clearance comparable to INH alone [104,105].

Central nervous system tuberculosis (CNS–*M.tb*) is among the most severe manifestations of TB in HIV-positive individuals, characterized by significant inflammation and oxidative stress. TBM is characterized by severe intracerebral inflammation driven by cytokine storms, with excessive production of TNF-α and IL-6 [18,19]. These contribute to neurological impairment and poorer outcomes in co-infected patients [24]. High-dose RIF has shown promise in reducing bacterial burden without worsening intracerebral inflammation [106]. However, the use of adjunctive corticosteroids such as dexamethasone has been found to reduce inflammation yet may interfere with rifampin’s bactericidal activity due to lower concentrations in the brain [106]. A 2023 cross-sectional study in Ethiopia found that advanced stages of HIV and the accompanying immunosuppression are significantly associated with a high incidence of CNS–*M.tb*, likely due to a breakdown in immune defenses and the BBB. This study highlights the urgent need for interventions, particularly in patients who had a delayed initiation of INH prophylaxis and cotrimoxazole preventive therapy, both of which were linked to an increased risk of CNS–*M.tb* [107].

The emergence of multidrug-resistant tuberculosis (MDR–TB) presents challenges in HIV-positive patients, where prolonged treatment regiments and drug–drug interactions exacerbate morbidity and mortality. Resistance to first-line drugs, such as INH and RIF, is often driven by genetic mutations in *M.tb*, including changes in mycolic acid synthesis and upregulation of rifampin tolerance mechanisms [108,109]. As stated earlier, deferring INH prophylaxis was identified as a significant predictor of CNS–*M.tb*, which may exacerbate challenges in managing drug-resistant strains of *M.tb* due to delayed control of the infection [107]. Current protocols involve an intensive phase of 5–7 months and a maintenance phase of 10–14 months [110]. Regiments typically include fluoroquinolones, bedaquiline, linezolid, cycloserine, and injectable aminoglycosides, which can penetrate the CNS effectively [111]. Adjunctive therapies, such as antioxidants like GSH, are also being explored for their ability to reduce oxidative stress, limit granuloma formation, and enhance host immune responses, potentially offering an additional layer of defense against drug-resistant *M.tb* [78]. These strategies highlight the importance of integrating innovative treatments to address the dual burden of drug resistance and co-infection in HIV-positive patients.

### 3.7. Preclinical and Clinical Studies Involving GSH Supplementation

The Synergistic Effects of the GSH Precursor, NAC and First-Line Antibiotics in the Granulomatous Response Against *M.tb* [112]. In a 2018 study, NAC, GSH precursor, supplementation with the first-line TB antibiotics (INH, RIF, and EMB) were investigated. The results of the study showed that compared to untreated controls there was a 5-log decrease in *M.tb* viability in those treated with NAC. Furthermore, when NAC was added as an adjunct to the suboptimal doses of the first-line TB antibiotics, there was significantly more clearance of *M.tb*, along with complete clearance of the INH + NAC and RIF + NAC treatment groups. The stability, size, and density of granulomas were also improved in the NAC, along with an increase in IFN-gamma, which allowed the bacteria to be better controlled and improved phagolysosomal acidification. NAC supplementation also reduced inflammatory cytokines such as IL-6. This study demonstrated that NAC has the potential to be an effective adjunct in *M.tb* treatment, specifically in those with HIV with GSH deficiency.

#### 3.7.1. Liposomal Glutathione Supplementation Mitigates Extrapulmonary Tuberculosis in the Liver and Spleen [113]

In a 2023 study, mice infected with *M.tb* were supplemented with L-GSH. The results of the study showed that L-GSH supplementation resulted in a decrease in oxidative stress. Supplementation also increases IFN-gamma and TNF-alpha and decreases IL-10. The most significant results of the study were that L-GSH supplementation was shown to reduce the bacterial load of extrapulmonary *M.tb*, particularly in the spleen and liver. This study shows that L-GSH supplementation could aid in the reduction in dissemination of *M.tb* and could potentially prevent CNS involvement.

#### 3.7.2. Liposomal Glutathione Helps to Mitigate *Mycobacterium tuberculosis* Infection in the Lungs [114]

In this 2022 study, mice were infected with *M.tb* to establish pulmonary TB, then the effects of L-GSH supplementation were analyzed. The results showed that there was a significant decrease in malondialdehyde (MDA), a marker of oxidative stress, in those who were supplemented with L-GSH. L-GSH supplementation also leads to decreased survival of *M.tb* in the lungs along with reduced inflammation. This study showed that L-GSH supplementation improved immune function in those with PTB. *M.tb* infection starts in the lungs then disseminates to other organs, by containing the bacilli before it spreads to the CNS, L-GSH supplementation could aid in the treatment of those with HIV–*M.tb* CNS co-infection by preventing dissemination.

#### 3.7.3. Effects of Glutathione Diminishment on the Immune Responses Against *Mycobacterium tuberculosis* Infection [115]

In this 2021 study, the effects of GSH reduction in mice were used to evaluate the impact on immune response in those infected with *M.tb*. Diethyl maleate was used to deplete GSH. The results of the study showed that decrease in the levels of GSH resulted in an increase in MDA levels. Also, those with decreased GSH had increases in levels of inflammatory cytokines, IL-1, and TNF-alpha, along with poor granuloma formation and increased *M.tb* survival. This study highlights the importance of GSH function in *M.tb* immune response. This further supports that GSH supplementation could be a potential effective adjunct in HIV–*M.tb* CNS infection, especially since those with HIV are known to have reduced GSH.

#### 3.7.4. Liposomes as Carriers for the Delivery of Efavirenz in Combination with Glutathione—An Approach to Combat Opportunistic Infections [116]

This 2022 study explored the use of liposomes to deliver the ART efavirenz (EFV) and GSH. The results of this study showed that the liposomal formulations improved uptake of EFV and GSH while also improving antioxidant activity. This study is significant because it shows the possibility of delivery methods of GSH and ARTs together to improve immune function. This could be especially useful in HIV–*M.tb* CNS co-infection, where the first-line treatments are ARTs and GSH could be added as an adjunct to improve the efficacy of not only HIV treatment but also TB innate immune response.

#### 3.7.5. Restoring Cytokine Balance in HIV-Positive Individuals with Low CD4 T Cell Counts [117]

In this 2017 double-blind study with 30 participants, L-GSH supplementation was given to those with HIV with CD4+ T cell counts < 350 mm^3^ subjects for 3 months. It was observed that the L-GSH supplementation group, had increases in cytokine levels of IL-12, IL-2, and IFN-gamma while also decreasing IL-6, IL-10, and free radicals. Additionally, in those receiving supplementation there was improved stabilization of TGF-b, IL-1, and IL-17. Furthermore, in the GSH supplementation group there were stabilized levels of free radicals in CD4+ T cells. In summary, L-GSH supplementation can help improve immune function by regulating inflammatory cytokine in those with HIV in order to boost immune function to reduce susceptibility to HIV–*M.tb* CNS co-infection.

#### 3.7.6. Liposomal Glutathione Supplementation Restores TH1 Cytokine Response to *Mycobacterium tuberculosis* Infection in HIV-Infected Individuals [101]

In this 2015 interventional study with 25 participants, individuals infected with HIV were given L-GSH supplementation over a period of 13 weeks. It was found that supplementation of L-GSH in People with HIV led to an increase in TH1 cytokines, such as IL-12 and IFN-gamma, and a decrease in TH2 cytokines, such as IL-4 and IL-5. Furthermore, supplementation also resulted in a decrease in free radicals and cytokines IL-10, TGF-b. It was also found that L-GSH led to improvement of control of *M.tb* infection. This study further demonstrated that L-GSH supplementation can aid in immune function and also control of *M.tb* in HIV-infected individuals to prevent CNS disease.

#### 3.7.7. Safety and Efficacy of N-Acetylcysteine in Hospitalized Patients with HIV-Associated Tuberculosis: An Open-Label, Randomized, Phase II Trial (RIPENACTB Study) [118]

In this 2020 study with 36 participants, hospitalized patients diagnosed with HIV–*M.tb* were randomized and treated with either anti-TB first-line therapy with or without NAC, a GSH precursor, and supplementation. The results of this study showed those in the NAC group had an improvement in CD4+ T cell count. Despite this, there were no significant differences in mortality rates between the NAC and no NAC group. This study shows that NAC could improve immune function through its ability to increase CD4+ T cell count in HIV–*M.tb* infected individuals. Even though there was no difference in survival rates between the two groups this could be due to several factors including the poor prognosis already associated with HIV–*M.tb* co-infection, NAC may be less effective compared to L-GSH supplementation, and the small sample size. Table 1 summarizes key studies on glutathione and its precursors in HIV–*M.tb* co-infection, detailing interventions, key findings, and clinical implications.

### 3.8. Implications for Future Research and Clinical Practice

Future research on HIV–*M.tb* co-infection should focus on addressing existing knowledge gaps through randomized clinical trials. These trials are essential for establishing efficacy of GSH supplementation in reducing microbial burden, enhancing immune function, and reducing inflammation in co-infected patients. Additionally, the development of CNS-directed GSH formulations is critical to overcome the challenges posed by BBB disruption and to ensure therapeutic levels of GSH in the CNS. Such formulations could be key in treating severe manifestations of co-infection, including TBM and HAND.

Integrating GSH into existing HIV and TB management protocols shows significant promise as an adjuvant therapy. GSH supplementation has the potential to enhance efficacy of HAART and first-line TB drugs, lower oxidative stress, and regulate inflammatory cytokine responses. In addition to infusion administration of glutathione, it has also been suggested that dietary supplementation with GlyNAC may be optimal since it contains two metabolic GSH precursors, i.e., N-acetyl-l-cysteine (NAC) and glycine (GLY) [119]. Its role as an adjunct could not only improve clinical outcomes but also reduce complications associated with IRIS and MDR–TB. Clinical guidelines should consider including GSH into routine care for co-infected patients, particularly in cases with CNS involvement. To ensure broad use, GSH therapies must be accessible and cost effective, particularly in settings where HIV and TB burden is the highest. Addressing logistics such as production, equitable distribution, and affordability will be important for GSH implementation into standard treatment protocols. Ethics should also guide clinical trials and implementation strategies, making sure there is inclusivity and prioritization of underserved populations.

## 4. Conclusions

GSH plays a key role in managing HIV–*M.tb* co-infection through its antioxidant and immunomodulatory properties. Evidence supports the additive effects of GSH in enhancing the efficacy of antibiotics and HAART, reducing inflammation, and improving immune recovery. These findings underline GSH’s potential as a complementary therapy to address the significant challenges posed by co-infection, including CNS involvement, drug resistance, and chronic inflammation. Despite promising findings, further research is essential to fully understand the therapeutic potential of GSH in HIV–*M.tb* co-infection. Innovative treatment strategies could include the development of formulations specific to the CNS and placing it into existing management. Addressing these points can lead to more effective and equitable care for coinfected individuals.

## Figures and Tables

**Table 1:** Results of studies on glutathione (GSH) and its precursors in HIV–*M.tb* co-Infection.

Study	Intervention	Key Findings	Implications
Teskey et al., 2018 [112]	NAC + first-line TB antibiotics	5-log decrease in *M.tb* viability with NAC supplementation. Improved granuloma stability. Reduced inflammatory cytokines (e.g., IL-6).	NAC improves antibiotic efficacy, enhances immune response, and reduces inflammation in HIV*-M.tb* co-infection.
Sasaninia et al., 2023 [113]	Liposomal GSH supplementation (L-GSH)	Reduced oxidative stress markers. Lower bacterial loads in spleen and liver. Increased IFN-γ and TNF-α levels.	L-GSH reduces dissemination of *M.tb* to extrapulmonary sites, potentially preventing CNS involvement.
Kachour et al., 2022 [114]	L-GSH supplementation	Decreased malondialdehyde (MDA). Lower *M.tb* survival in lungs. Reduced inflammation.	L-GSH limits pulmonary TB progression and may prevent CNS dissemination in HIV-*M.tb* co-infection.
Cao et al., 2021 [115]	GSH depletion model	Increased inflammatory cytokines (IL-1, TNF-α). Poor granuloma formation. Increased M.tb survival.	Demonstrates the critical role of GSH in immune defense and the potential benefits of supplementation.
Kenchappa et al., 2022 [116]	Liposomal delivery of Efavirenz + GSH	Enhanced drug uptake. Improved antioxidant activity.	Liposomal formulations could combine GSH and ARTs for enhanced efficacy against HIV-*M.tb* CNS infection.
Valdivia et al., 2017 [117]	L-GSH in HIV with CD4 < 350/mm³	Increased Th1 cytokines (IL-2, IL-12, IFN-γ). Reduced IL-6, IL-10. Stabilized free radicals.	GSH supplementation improves immune recovery and reduces inflammation in HIV-*M.tb* co-infection.
Ly et al., 2015 [101]	L-GSH in HIV-infected individuals	Increased Th1 cytokines. Reduced free radicals and Th2 cytokines. Improved *M.tb* control.	Highlights L-GSH’s role in controlling *M.tb* infection and enhancing immune function in HIV-positive individuals.
Safe et al., 2020 (RIPENACTB Study) [118]	NAC in HIV-associated TB	Improved CD4+ T-cell count. No significant difference in mortality.	NAC improves immune recovery but requires further investigation for survival benefits in severe cases.
Tuell et al., 2024 [119]	GlyNAC supplementation	Combined NAC and glycine improve GSH levels. Mitigates oxidative stress.	Dietary supplementation with GSH precursors offers an accessible approach for enhancing immune responses.
Ruiz-Bedoya et al., 2022 [107]	High-dose rifampin	Lowered bacterial burden. Did not exacerbate inflammation.	Adjunctive rifampin therapy shows promise but needs integration with anti-inflammatory strategies like GSH.

## Data Availability

Data available in references and Supplementary Materials [120].

## Supplementary Materials

The following supporting information can be downloaded at: https://www.mdpi.com/article/10.3390/v17010127/s1. File: PRISMA 2020 Checklist.
